# SOX2 Is a Potential Diagnostic Biomarker and Anticancer Target in Cutaneous Squamous Cell Carcinoma in Dystrophic Epidermolysis Bullosa: A Case Series Study

**DOI:** 10.1016/j.xjidi.2025.100417

**Published:** 2025-09-22

**Authors:** Clara Harrs, Marieke Bolling, Peter van den Akker, Bart Koopman, Barbara Horváth, Gilles Diercks, Léon van Kempen

**Affiliations:** 1 Center for Blistering Diseases, Department of Pathology, University Medical Center Groningen, University of Groningen, Groningen, The Netherlands; 2 Center for Blistering Diseases, Department of Dermatology, University Medical Center Groningen, University of Groningen, Groningen, The Netherlands; 3 Center for Blistering Diseases, Department of Genetics, University Medical Center Groningen, University of Groningen, Groningen, The Netherlands; 4 Department of Pathology, Maastricht University Medical Center+, University of Maastricht, Maastricht, The Netherlands

**Keywords:** Anticancer target, Biomarker, Cutaneous squamous cell carcinoma, Epidermolysis bullosa, SOX2

## Abstract

A serious complication in epidermolysis bullosa (EB), particularly in recessive dystrophic EB, is the development of aggressive cutaneous squamous cell carcinoma with a high risk for metastasis and poor survival outcomes. The current standard of care is insufficient, and there is a critical need for safe and effective management options for this life-threatening complication in EB. Moreover, the pathophysiologic processes behind the aggressiveness of EB-associated cutaneous squamous cell carcinoma (EB-cSCC) remain elusive. Especially little is known about genetic drivers specific for EB-cSCC, and information about the molecular profile of these highly malignant tumors could lead to novel insights about the underlying pathogenesis. In addition, EB-associated pseudoepitheliomatous hyperplasia (EB-PEH) is difficult to distinguish from EB-cSCC histologically and could be a premalignant precursor of EB-cSCC. Therefore, the aim of this study was to conduct gene expression profiling to detect potentially diagnostic biomarkers and anticancer targets in EB-cSCC and explore the potential role of EB-PEH in the tumorigenesis of EB-cSCC. We performed mRNA expression analysis and immunohistochemistry on formalin-fixed, paraffin-embedded tissue samples of EB-cSCCs (n = 8), non–EB-cSCCs (n = 10), and EB-PEHs (n = 7). The results revealed upregulation of *SOX2* mRNA expression in EB-cSCCs compared with that in non–EB-cSCCs, albeit in this study, the sample size was small, and cases and controls were not age matched, which may limit interpretation of our findings. In addition, immunohistochemical staining showed increased SOX2 protein expression in EB-cSCCs compared with that in non–EB-cSCCs. SOX2 protein expression was also observed in EB-PEH, with 1 case showing a transition from SOX2-negative to SOX2-positive cells, indicating the possible initiation of an EB-cSCC in situ. Further validation of the study results, including mechanistic studies, is needed before the utility of SOX2 as a biomarker can be assessed. However, these findings propose that SOX2 may play a role in the development of aggressive EB-cSCC and could serve as a potential biomarker of progression from benign EB-PEH to EB-cSCC.

## Introduction

The various forms of epidermolysis bullosa (EB) comprise a heterogeneous group of rare genetic mucocutaneous fragility disorders, with an incidence ranging from 20 to 41 cases per million life births, caused by sequence variants in genes encoding for structural proteins of the basement membrane zone (Baardman et al, 2021; Fine, 2016; Has et al, 2020). EB is characterized by development of blisters and chronic wounds, and the clinical spectrum ranges from phenotypes with mild localized lesions to severe disabling forms with extensive blistering, fibrosis, and extracutaneous involvement (Fine et al, 2014). Depending on the level of blister formation within the skin, EB is classified into 4 main types and 34 subtypes on the basis of inheritance, molecular features, and clinical characteristics (Has et al, 2020).

Pseudoepitheliomatous hyperplasia (PEH), commonly regarded as a benign epithelial proliferation, is often encountered in chronic wounds of patients with EB. PEH may be challenging to histologically differentiate from cutaneous squamous cell carcinoma (cSCC) in patients with EB (Mellerio et al, 2016), which may implicate PEH as a potential cSCC precursor in EB.

Early-onset aggressive cSCC is a serious complication in EB, with a high tendency for metastasis and local relapse. In patients with the recessive dystrophic EB (RDEB), which is caused by the loss of type VII collagen, EB-associated cSCCs (EB-cSCCs) behave aggressively and are the leading cause of morbidity and death (Castelo et al, 2019; Fine et al, 2009; Harrs et al, 2022; Kim et al, 2018; Robertson et al, 2021). A nationwide study within the Dutch Epidermolysis Bullosa Registry was conducted to analyze clinical outcomes and tumor characteristics of EB-cSCCs. Paradoxically, EB-cSCCs have an aggressive nature despite the absence of most histopathological risk factors, such as poor differentiation and lymphovascular invasion (Harrs et al, 2022).

The current standard of care consists of regular screening and wide local excision. However, management options are inadequate, which is underscored by a high percentage of incomplete resected tumors and poor survival outcomes, including a median survival <5 years and median age at death of 38 years in patients with RDEB (Harrs et al, 2022; Kim et al, 2018; Mellerio et al, 2016; Paganelli et al, 2022; Robertson et al, 2021). Hence, there is an urgent demand for safe and effective disease control, especially with curative and preventive intention.

EB-cSCCs are clearly more aggressive than sporadic cSCCs, which is caused by UVR, but the underlying pathogenesis is not fully understood. It is surmised that the aggressive nature of the EB-cSCCs may be more dependent on a “tumor-prone” environment than on the tumor itself. This hypothesis is supported by the growing evidence that the repetitive wound and scar formation in RDEB leads to extensive fibrosis, creating a permissive tumor microenvironment (Dayal et al, 2021; Guerra et al, 2017; Küttner et al, 2013; Ng et al, 2012; Tartaglia et al, 2021).

The exact molecular mechanisms underlying this process are not known, but the loss of type VII collagen seems to increase TGF-β signaling, leading to tumor-promoting changes in the extracellular matrix (Küttner et al, 2013; Ng et al, 2012). In addition, it is postulated that immune dysfunction in EB, characterized by reduced immune surveillance and increased activation of proinflammatory pathways with concomitant immune exhaustion, may promote tumorigenesis in RDEB (Bernasconi et al, 2021; Filoni et al, 2022; South et al, 2023).

Little is known about the genetic drivers specific for EB-cSCC because only a few studies on molecular profiling of EB-cSCCs have been published. EB-cSCCs probably arise from mutagenic processes that are different from those prevalent in UV-induced cSCC because a significantly lower number of mutational signatures associated with UVR was detected in EB-cSCC (Cho et al, 2018). Interestingly, it was hypothesized that dominant mutational signatures in EB-cSCCs were associated with repetitive tissue damage because a high rate of mutations that emerged in RDEB-associated cSCCs was driven by specific gene editors (APOBEC [apolipoprotein B mRNA-editing enzyme catalytic polypeptide‒like] deaminases), which particularly seemed to show activity in areas of chronic wounds (Cho et al, 2018). The molecular etiology of the tumorigenicity in EB and the influence of different intrinsic and extrinsic factors, such as microenvironmental remodeling or dysfunctional immunity, are areas of ongoing study. Therefore, more information about the expression profile of cancer and immune response–related genes in EB-cSCCs could lead to novel insights about the pathogenesis of these highly malignant tumors.

To gain better insights into the pathophysiologic processes and help to identify potential diagnostic biomarkers and anticancer targets, we performed a gene expression analysis of EB-cSCCs and compared them with non–EB-cSCCs and histologically proven PEH lesions of the same patients with EB.

## Case Series Report

Formalin-fixed, paraffin-embedded (FFPE) tissues of 8 patients with severe RDEB with preferably well-differentiated cSCCs, known with a history of local recurrence and/or metastasis, were selected from the Dutch Epidermolysis Bullosa Registry for gene expression profiling. Of each patient with RDEB (average age at diagnosis = 30.7 ± 11.3 years), at least 1 cSCC was included. In addition, if available, FFPE tissues containing PEHs of the same patients with RDEB were selected (EB-associated PEH [EB-PEH]), which preferably developed on the same location as the EB-cSCC. For the control group, FFPE tissues of 10 elderly patients (average age at diagnosis = 80.7 ± 11.1 years) with well-differentiated cSCC of sun-exposed skin were chosen (UV-induced cSCC).

A total of 26 FFPE tissues (9 EB-cSCC, 7 EB-PEH, 10 UV-induced cSCC) were retrieved from the Pathology biobank of the University Medical Center Groningen. To measure differences in mRNA expression levels, a NanoString mRNA expression analysis with the nCounter PanCancer IO 360 Panel was performed (NanoString nCounter Technologies).

**Figure 1:**
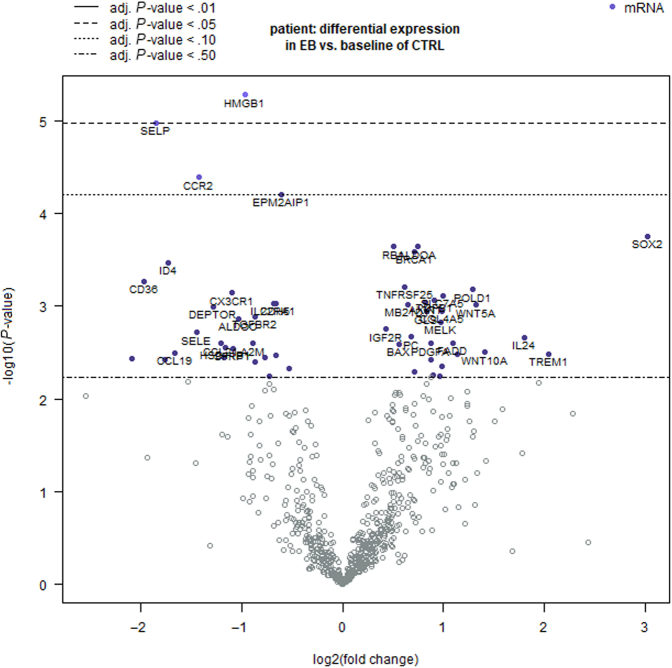
**Volcano plot: differential expression analysis of EB-cSCC compared with the UV-induced cSCC (baseline).** The x-axis depicts the log_2_ fold change, and the y-axis depicts the −log_10_ (*P*-value). The horizontal lines indicate various FDR-adjusted *P*-value thresholds. The genes are colored purple if the resulting FDR-adjusted *P* < .50. Statistically significant genes (FDR-adjusted *P* < .05) are depicted above the highest dotted line (- - -). A total of 40 genes with the lowest FDR-adjusted *P*-values are labeled in the plot (cut-off threshold: FDR-adjusted *P* > .375). Highly differentially expressed genes (log_2_ fold change > 2.0 or < −2.0) fall to either side of the x-axis. The differential gene expression analyses indicated significantly decreased expression of *HMGB1* and *SELP* (*P*-value and FDR-adjusted *P* < .05) and increased expression of *SOX2* (*P* < .05; FDR-adjusted *P* = .148) in EB-cSCC relative to those in UV-induced cSCC. *HMGB1* and *SELP* both displayed a log_2_ fold change <2.0, whereas *SOX2* was highly differentially expressed in EB-cSCC compared with that in UV-induced cSCC with a log_2_ fold change of 3.02 (albeit not significantly different, FDR-adjusted *P =* .148). adj. p-value, adjusted P-value; cSCC, cutaneous squamous cell carcinoma; EB-cSCC, epidermolysis bullosa–associated cutaneous squamous cell carcinoma; FDR, false discovery rate.

A false discovery rate (FDR)–adjusted *P* < .05 was categorized as a statistically significant expression and a log_2_ fold change >2.0 or < −2.0 as a highly differential expression. Differential gene expression indicated, among others, significantly decreased expression of *HMGB1* and *SELP* (FDR*-*adjusted *P* < .05) and increased expression of *SRY* (known as *SOX2*, in EB-cSCC relative to UV-induced cSCC) (FDR-adjusted *P* = .148) (Figure 1). However, *HMGB1*, *SELP*, and other decreased expressed genes showed a log_2_ fold change <2.0, indicating that these genes do not demonstrate strong differential expression between EB-cSCC and the UV-induced cSCC. In contrast, a log_2_ fold change of 3.02 revealed that *SOX2* was highly differentially expressed in EB-cSCC compared with that in the UV-induced cSCC. This suggests that the upregulation of *SOX2* could be a mechanism in the pathogenesis of EB-cSCC, although *SOX2* was not significantly expressed on the basis of the FDR-adjusted *P*-value. Regarding EB-PEH, no significant differences in gene expression compared with EB-cSCC were detected, and the average *SOX2* mRNA expression level was low.

To further explore the role of *SOX2* mRNA expression, immunohistochemistry was performed to determine the effect on differential gene expression on protein expression. A semiqualitative analysis of the SOX2 staining pattern of all 26 FFPE tissue slides and a semiautomatic quantitative analysis for the difference in SOX2 protein expression between EB-cSCC and UV-induced cSCC were conducted.

**Figure 2:**
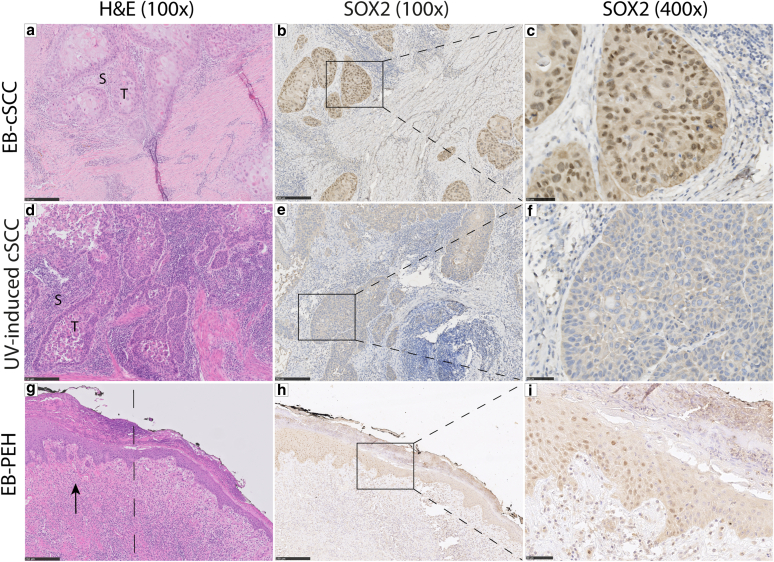
**Nuclear SOX2 expression in EB-cSCC, UV-induced cSCC, and EB-PEH.(a)** Representative H&E staining of a well-differentiated EB-cSCC, characterized by sharply demarcated nests of squamous epithelium (denoted as T) surrounded by stroma (denoted as S). (**b, c)** Positive nuclear SOX2 expression in the EB-cSCC with especially SOX2-positive tumor cells along the tumor–stroma interface. **(d)** Representative H&E staining of a well-differentiated UV-induced cSCC, with squamous epithelium (denoted as T) surrounded by stroma (denoted as S). **(e, f)** Absent (negative) nuclear SOX2 expression in the UV-induced cSCC. (**g)** The EB-PEH shows variable epidermal hyperplasia with focally increase of hyperplasia (dotted line) and onset of cytonuclear atypia suspicious for a (developing) EB-cSCC in situ (black arrow). **(h, i)** In the region morphologically suspicious for a cSCC in situ, a gradual transition from negative to positive SOX2 expression in all epidermal layers beside the stratum corneum is seen. Original magnification: (**a, b, d, e, g, h**): ×100; (**c, f, i**): ×400. Bar = (**a, b, d, e, g, h**) = 250 μm and (**c, f, i**) = 50 μm. cSCC, cutaneous squamous cell carcinoma; EB-cSCC, epidermolysis bullosa–associated cutaneous squamous cell carcinoma; EB-PEH, epidermolysis bullosa–associated pseudoepitheliomatous hyperplasia.

For the semiqualitative assessment, nuclear staining of SOX2 was considered positive, and all 26 FFPE tissue slides were categorized having positive or absent SOX2 nuclear expression. Six of 9 EB-cSCC (67.6%) showed a positive SOX2 expression in especially tumor cells along the tumor–stroma interface (TSI) (Figure 2a–c), whereas the UV-induced cSCC demonstrated absent nuclear SOX2 expression (0 of 10) (Figure 2d–f). In contrast to the low average *SOX2* mRNA expression in the EB-PEH cohort, subtle SOX2 protein expression could be observed in 3 of 7 EB-PEH (42.8%). The first and second positive cases were biopsies with limited available tissue, but a diffuse positive expression in the epidermis was seen. Strikingly, in the third positive case, an excision, a gradual transition from SOX2-negative to SOX2-positive epithelial cells was detected in a region morphologically suspicious for a cSCC in situ (Figure 2g–i).

In the semiautomatic quantitative analysis, both the percentage of SOX2-positive tumor cells and the Histochemical score (H-score) were calculated. The H-score reflects the combined effect of the proportion of positive cells and their staining intensity. The SOX2 protein expression showed in the EB-cSCC group a median percentage of 12.12% SOX2-positive cells (minimum = 2.03%, maximum= 57.36%, interquartile range [IQR] = 7.68–35.08%), whereas the median percentage in UV-induced cSCC was 3.83% (minimum = 0.88%, maximum = 11.31%, IQR = 2.12–6.24%). On the basis of the Wilcoxon rank sum exact test, EB-cSCC showed a significantly higher percentage of SOX2-positive tumor cells than UV-induced cSCC (W = 75, *P* = .01327).

Concerning the H-score of the SOX2 protein expression, the median in EB-cSCC was 14.0 (minimum = 2.3, maximum = 83.2, IQR = 9.1–48.5), whereas in UV-induced cSCC, the median H-score was 3.8 (minimum = 0.9, maximum = 12.5, IQR = 2.4–6.7). Comparable with the percentage of SOX2-positive tumor cells, EB-cSCC exhibited a significantly higher H-score than UV-induced cSCC (W = 76, *P* = .01013).

**Figure 3:**
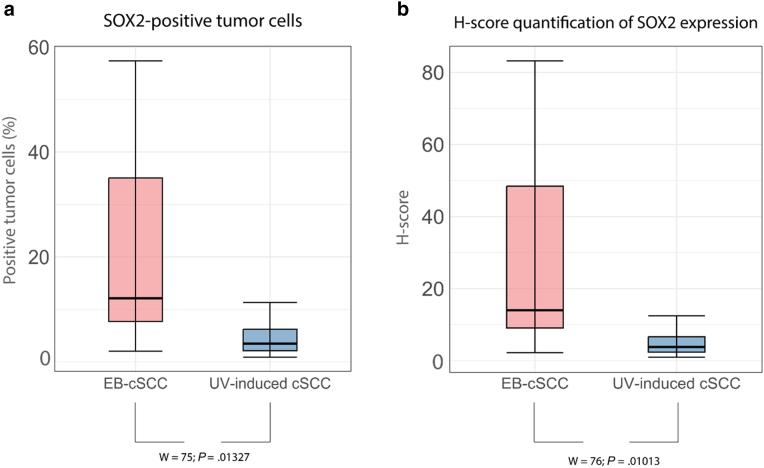
**Distribution of percentage of SOX2-positive tumor cells (panel a) and H-score of SOX2 expression (panel b) between EB-cSCC and UV-induced cSCC based on semiautomatic quantitative analysis.(a)** The median percentage of SOX2-positive tumor cells in EB-cSCC was significantly higher than in UV-induced cSCC (W = 75, *P* = .01327) (EB-cSCC [median = 12.12%, minimum = 2.03%, maximum = 57.36%, IQR = 7.68–35.08%]; UV-induced cSCC [median = 3.83%, minimum = 0.88%, maximum = 11.31%, IQR = 2.12–6.24%]). **(b)** The median H-score for SOX2 expression in EB-cSCC was significantly higher than in UV-induced cSCC (W = 76, *P* = .01013) (EB-cSCC [median = 14.01, minimum = 2.25, maximum = 83.17, IQR = 9.10–48.46]; UV-induced cSCC [median = 3.83, minimum = 0.99, maximum = 12.51, IQR = 2.41–6.66]). cSCC, cutaneous squamous cell carcinoma; EB-cSCC, epidermolysis bullosa–associated cutaneous squamous cell carcinoma; H-score, Histochemical score; IQR, interquartile range.

The distribution of the percentage of SOX2-positive tumor cells and the H-score between both groups were visualized in Figure 3.

Clinical and histological characteristics, along with the results of the semiqualitative assessment and the semiautomatic quantitative analysis of the SOX2 protein expression, are described in Table 1.

**Table 1:** Clinical Characteristics and Results of Immunohistochemical Analysis in EB-cSCC, EB-PEH, and UV-induced cSCC

Patient ID	Group	EB Type	Excision/biopsy	Age at Diagnosis, y	Sex	Location of cSCC	Histological Characteristics	SOX2 IHC Staining Pattern (Semiqualitative Analysis)	Percentage SOX2-Positive Tumor Cells (Semiautomated Analysis)	H-score for SOX2 IHC Expression (Semiautomated analysis)
EB-1	EB-cSCC	RDEB severe	Excision	38	M	Plantar aspect of the foot (R)	Well-differentiated	No detectable nuclear staining (negative)	3.06%	3.8
EB-2	EB-cSCC	RDEB severe	Excision	34	M	Central neck (dorsal)	Well-differentiated	Tumor cells with positive staining at tumor–stroma interface (positive)	12.12%	14
EB-3	EB-cSCC	RDEB severe	Excision	28	F	Hand (R)	Well-differentiated	Tumor cells with positive staining at tumor–stroma interface (positive)	57.36%	83.2
EB-4	EB-cSCC	RDEB severe	Excision	42	M	Hand with 'coccon' deformity (L)	Well-differentiated, Perineural invasion	Tumor cells with positive staining at tumor–stroma interface (positive)	35.08%	48.5
EB-5.1	EB-cSCC	RDEB severe	Excision	26	M	Hand (R)	Well-differentiated	Tumor cells with positive staining at tumor–stroma interface (positive)	14.46%	17.3
EB-5.2	EB-cSCC	RDEB severe	Excision	31	M	Hand (L): thenar (hand highly misformed)	Well-differentiated	No detectable nuclear staining (negative)	2.03%	2.3
EB-6	EB-cSCC	RDEB severe	Excision	22	M	Hand (R)	Well-differentiated	No detectable nuclear staining (negative)	7.68%	9.1
EB-7	EB-cSCC	RDEB severe	Excision	34	M	Heel/malleolus (L)	Well-differentiated	Tumor cells with positive staining at tumor–stroma interface (positive)	36.68%	53.5
EB-8	EB-cSCC	RDEB severe	Excision	22	M	Forearm (L)	Moderate differentiated, angioinvasion	Tumor cells with positive staining at tumor–stroma interface (positive)	10.56%	12.6
UV-1	UV-induced cSCC	—	Excision	92	F	Hand (L)	Well-differentiated	No detectable nuclear staining; diffuse nonspecific cytoplasmic background staining (negative)	11.31%	∗12.5∗
UV-2	UV-induced cSCC	—	Excision	80	M	Hand (R)	Well-differentiated	No detectable nuclear staining (negative)	2.23%	2.5
UV-3	UV-induced cSCC	—	Excision	69	M	Hand (R)	Well-differentiated	No detectable nuclear staining (negative)	2.17%	2.4
UV-4	UV-induced cSCC	—	Biopsy	82	M	Hand (L)	Well-differentiated	No detectable nuclear staining; Diffuse nonspecific cytoplasmic background staining (negative)	6.39%	6.8
UV-5	UV-induced cSCC	—	Excision	87	M	Hand (R)	Well-differentiated	No detectable nuclear staining (negative)	4.73%	5.2
UV-6	UV-induced cSCC	—	Excision	74	F	Hand (L)	Well-differentiated	No detectable nuclear staining (negative)	6.5%	7.4
UV-7	UV-induced cSCC	—	Excision	70	M	Hand (R)	Well-differentiated	No detectable nuclear staining (negative)	5.78%	6.3
UV-8	UV-induced cSCC	—	Excision	90	M	Hand (R)	Well-differentiated	No detectable nuclear staining (negative)	2.11%	2.4
UV-9	UV-induced cSCC	—	Excision	81	F	Foot (R)	Well-differentiated	No detectable nuclear staining (negative)	1.37%	1.5
UV-10	UV-induced cSCC	—	Biopsy	82	F	Hand (side not known)	Well-differentiated	No detectable nuclear staining (negative)	0.88%	0.9
EB-1	EB-PEH	RDEB severe	Biopsy	38	M	Plantar aspect of the foot (R)	N/A	No detectable nuclear staining (negative)	N/A	N/A
EB-2	EB-PEH	RDEB severe	Biopsy	32	M	Upper arm (L)	N/A	No detectable nuclear staining (negative)	N/A	N/A
EB-3	EB-PEH	RDEB severe	Biopsy	6	F	Knee (R)	N/A	No detectable nuclear staining (negative)	N/A	N/A
EB-4	EB-PEH	RDEB severe	Biopsy	41	M	Hand with 'coccon' deformity (L)	N/A	No detectable nuclear staining (negative)	N/A	N/A
EB-5	EB-PEH	RDEB severe	Excision	32	M	Forearm (L)	N/A	Gradual transition from SOX2-negative to positive nuclear SOX2 expression in a region morphologically suspicious for a cSCC (in situ) (positive)	N/A	N/A
EB-6	EB-PEH	RDEB severe	Biopsy	15	M	Upper leg (R)	N/A	Diffuse positive nuclear expression in all epidermal layers beside stratum corneum (positive)	N/A	N/A
EB-7	EB-PEH	RDEB severe	Biopsy	33	M	Heel (L)	N/A	Diffuse positive nuclear expression in all epidermal layers beside stratum corneum (positive)	N/A	N/A

Abbreviations: cSCC, cutaneous squamous cell carcinoma; EB, epidermolysis bullosa; EB-cSCC, epidermolysis bullosa–associated cutaneous squamous cell carcinoma; EB-PEH, epidermolysis bullosa–associated pseudoepitheliomatous hyperplasia; F, female; H-score, Histochemical score; ID, identification; IHC, immunohistochemistry; L, left; M, male; N/A, not available; R, right; RDEB, recessive dystrophic epidermolysis bullosa.

## Discussion

Analyses of the transcriptome in EB-PEH, EB-cSCC, and UV-induced cSCC reveal a possible role for SOX2 in EB-cSCC. SOX2 is a well-characterized transcription factor that plays a crucial role in stem cell development and cross-talks with various signaling pathways that regulate cell proliferation, survival, and tumorigenesis (Boumahdi et al, 2014). It was postulated that overexpression of SOX2 induces cancer stemness, tumor invasion, and drug resistance and is associated with poor survival in different types of cancers (Hou et al, 2015; Tang et al, 2013; Zhang et al, 2020).

On the basis of the increased *SOX2* gene expression and the significant higher percentage of SOX2-positive cells and higher H-score in EB-cSCC than in the UV-induced cSCC, we suggest that SOX2 could also have a key role in the tumor initiation and aggressive nature of EB-cSCC.

Although the median percentage of SOX2-positive tumor cells in UV-induced cSCC is low (3.83%), all UV-induced cSCCs were defined as having absent nuclear SOX2 staining in the semiqualitative assessment. One reason for the difference in results between the immunochemical analyses could be nonspecific cytoplasmic staining in tumor cells, which may be falsely interpreted as nuclear SOX2 localization, leading to false positives in the semiautomatic analysis. In addition, semiautomatic digital scoring of oral squamous cell carcinoma (SCC), which shares common histological features with cSCC, categorized SOX2 protein expression as 'absent/low' or 'high' on the basis of H-score cut-off values (absent/low SOX2 expression: H-scores of 0–11; high SOX2 expression: H-score ≥ 11) (Steen et al, 2025). According to these H-score cut-off values, UV-induced cSCC demonstrates absent/low SOX2 protein expression (median H-score = 3.8), which aligns with the results of the semiqualitative assessment.

In contrast to this observation in our UV-induced cSCC group, SOX2 expression has been described in cSCC previously. SOX2-positive tumor cells in primary human SCC, including cSCCs, were especially seen at the TSI (Siegle et al, 2014), which is in line with the SOX2 expression pattern in our EB-cSCC cohort (Figure 2b and c). In a murine cSCC model of the same study, SOX2 promoted the expansion of so-called tumor-initiating cells along the TSI, which have the ability to self-renew, give rise to differentiated cells, and form new tumors (Siegle et al, 2014). Because SOX2 seems to be an important regulator of cancer stem cell function and of tumor-initiating potential in sporadic cSCCs, we surmised that SOX2 expression in EB-cSCCs can contribute to its aggressive phenotype. Differences in the study population might explain why we did not observe pronounced SOX2 expression in the UV-induced cSCC as opposed to the findings in the study mentioned earlier (Siegle et al, 2014). We only selected well-differentiated cSCCs in elderly individuals to match with the histological appearance of EB-SCC (Table 1). Well-differentiated cSCCs in non-EB patients are associated with a low risk of metastasis, whereas the preceding study also included head and neck, anogenital, and internal SCCs with moderate and poorly differentiation (Siegle et al, 2014). In particular, high-grade head and neck SCCs are known to display aggressive behavior with a high risk of recurrence and an increased metastatic potential (Faraji et al, 2023). Therefore, SOX2 expression appears to be associated with high-risk SCCs, including well-differentiated EB-cSCC despite the lack of histopathological prognostic factors of aggressiveness.

Although PEH is defined as a reactive epithelial proliferation, we hypothesize that EB-PEH could be a potential premalignant precursor in EB owing to the SOX2 expression in 3 EB-PEH. Especially in the third EB-PEH case that was presented, a gradual transition from SOX2-negative to SOX2-positive epithelial cells in a region suspicious for a cSCC in situ was seen, indicating the possible initiation of an EB-cSCC (Figure 2g–i). Hence, SOX2 could also be a potential indicator for the detection of developing EB-cSCC (in situ).

The underlying mechanism behind the upregulated SOX2 expression in EB-cSCC remains a subject for further study. A possible explanation could be that the increased TGF-β signaling in the profibrotic microenvironment of EB skin leads to SOX2 overexpression. This hypothesis is based on the results in several studies with cell lines, including cSCC, in which it was shown that TGF-β signaling can regulate SOX2 expression through the TGF-β/SMAD signaling pathway (Li et al, 2019; Liu et al, 2018; Weina et al, 2016). In our cohort, we did not detect significant over or underexpression (FDR-adjusted *P* < .5) or substantial differential expression (log_2_ fold change < 2.0) of *TGFB1*, *TGFB2*, *TGFB3*, *TGFBR1*, *TGFBR2*, *SMAD5*, and others in EB-cSCC compared with those in UV-induced cSCC. This does not exclude the involvement of TGF-β signaling because effects on protein activation without differential gene expression were not measured. For instance, the receptor phosphorylation of the TGF-β receptors, which is a critical first step in the TGF-β signaling cascade, cannot be detected with mRNA expression analysis. Therefore, complementary protein expression analysis in the future could be useful for determining whether the levels of proteins associated with the TGF-β/SMAD signaling pathway significantly differ between EB-cSCC and UV-induced cSCC.

Considering the potential role of SOX2 in tumor initiation of the aggressive EB-cSCC, targeting SOX2 presents a promising approach for anticancer therapy. However, in ovarian cancer cell lines, SOX2 is a contextual and contrastingly regulated signaling node downstream of TGF-β (Shonibare et al, 2022). This implies that SOX2 biology observed in 1 system cannot be generalized. Nevertheless, ongoing research on SOX2-targeting approaches may be relevant for EB-cSCC as well (Ji et al, 2018; Liu et al, 2020; Taniguchi et al, 2017; Yin et al, 2019; Yokota et al, 2017).

Our study is not without limitations. Because EB is a rare disease, our study is limited by a small sample size, making it difficult to draw firm conclusions. In addition, the cases and controls could not be age matched owing to age-related onset of cSSC in EB and controls. Age may therefore be a confounding factor that could not be corrected for. Owing to the scarcity of samples, the differential gene expression profiles presented in this work lack statistical power with an increased risk of type II errors (false negatives) and potential overestimation of fold changes.

Moreover, given the small sample size and large number of data points, the likelihood that genes are not significantly expressed with an FDR-adjusted *P* < .05 is higher, underscoring the importance of independent validation.

However, verification of differentially expressed genes with an orthogonal method, namely immunohistochemical analyses, demonstrated that differential *SOX2* expression at the mRNA level is reflected in SOX2 protein expression.

Therefore, although our findings warrant further validation, including mechanistic studies, before SOX2 can be used as a biomarker, the results of this case series study may contribute to a better understanding of the underlying mechanisms behind the aggressive nature of EB-cSCC. In addition, the findings may help fuel hypotheses to understand the underlying pathophysiology of this life-threatening complication in EB.

## Materials and Methods

### Study design

This case series investigated differences in gene expression between EB-cSCC, EB-PEH, and UV-induced cSCC in a total of 8 patients with EB and 10 control patients and evaluated the effect of the upregulation of *SOX2* expression on protein expression. The study protocol was reviewed and approved by the assigned Central and Local ethics Review Boards. Only deidentified rest material (FFPE tissues) of the Pathology biobank in the University Medical Center Groningen was collected for the study retrospectively. Therefore, no informed consent of the patients was required, and the study was not considered research subject to the Medical Research Involving Human Subjects Act.

### NanoString mRNA expression analysis

#### RNA extraction

For the mRNA expression analysis, total RNA was isolated from the 5-μm-thick 26 FFPE tissues using the RNeasy FFPE kit according to suppliers’ instruction (Qiagen). Total RNA was quantified with Qubit (Thermo Fisher Scientific).

#### NanoString gene expression profiling

The Nanostring nCounter PanCancer IO 360 Panel (NanoString nCounter Technologies) was used to measure mRNA gene expression levels between the experimental groups, including 770 cancer and immune response–related genes. The probes of the panel were hybridized with 100 ng RNA overnight in a thermocycler at 65 °C with a heated-lid at 70 °C. The RNA–probe complexes were loaded on an nCounter cartridge and hybridized, washed, and read on a nCounter SPRINT platform according to the suppliers’ instructions (NanoString nCounter Technologies).

#### Data analysis

Quality control and data normalization were performed with the nSolver Analysis Software (version 4.0) (NanoString nCounter Technologies) to correct for differences in hybridization efficiency using the respective control probes. The threshold for background expression was based on the geometric mean expression of all negative control probes plus 3 SDs. Genes with an expression higher than the mean expression of all genes above background plus 3 SDs were identified as very highly expressed genes. Subsequently, whole-content normalization was performed, including all but the highly expressed genes that were expressed above background.

For the differential expression analysis, R-based nSolver Analysis Software (version 4.0) (NanoString Technologies) with the Advanced analysis plugin (version 2.0.1.34) was used. A negative binomial regression model was applied for the estimation of the differential expression of each target gene between the experimental groups (depicted as the log_2_ fold change). FDR-adjusted *P*-values were calculated using the Benjamini–Yekutieli procedure embedded within the nSolver software, and an FDR-adjusted *P* < .05 was categorized as statistically significant. In addition, the 95% confidence interval for the log fold change was calculated, and a log_2_ fold change >2.0 or < −2.0 was categorized as highly differentially expressed.

The differential gene expression data are visualized in a volcano plot (Figure 1). For visualization of the differential gene expression between EB-cSCC and UV-induced cSCC, the software automatically colored genes in the volcano plot purple if the FDR-adjusted *P*-value was <.50 and labeled 40 genes with the lowest FDR-adjusted *P*-values (cut-off threshold: FRD-adjusted *P* > .375).

### Immunohistochemistry

Immunohistochemical staining was performed on the FFPE tissue of the EB-cSCC, EB-PEH, and UV-induced cSCC. The FFPE tissues were cut at a thickness of 5 μm using a microtome and underwent deparaffinization with xylene and rehydration with graded ethanol. Antigen retrieval was performed by treating the sections with a Tris-EDTA buffer (10 mM Tris/1 mM EDTA, pH 9.0) for 15 minutes at cooking temperature in the microwave. The sections were then washed with PBS, and endogenous peroxidase activity was blocked using 0.3% hydrogen peroxidase for 30 minutes and washed again with PBS. Subsequently, sections were incubated for an hour at room temperature with a validated primary SOX2 antibody (polyclonal rabbit, 1:1000, Seven Hills Bioreagents, number WRAB-1236) diluted in 1% BSA/PBS. After washes, secondary (polyclonal goat antirabbit, 1:1000, Agilent Dako, number P0448) and tertiary (polyclonal rabbit antigoat, 1:1000, Agilent Dako, number P0449) antibodies were applied for 30 minutes, diluted in PBS-1% BSA with 1% human AB-serum. Positive staining was visualized using Vector NovaRED substrate (SK-4800, Vector Laboratories). The sections were counterstained with hematoxylin, followed by dehydration with graded ethanol.

**Figure 4:**
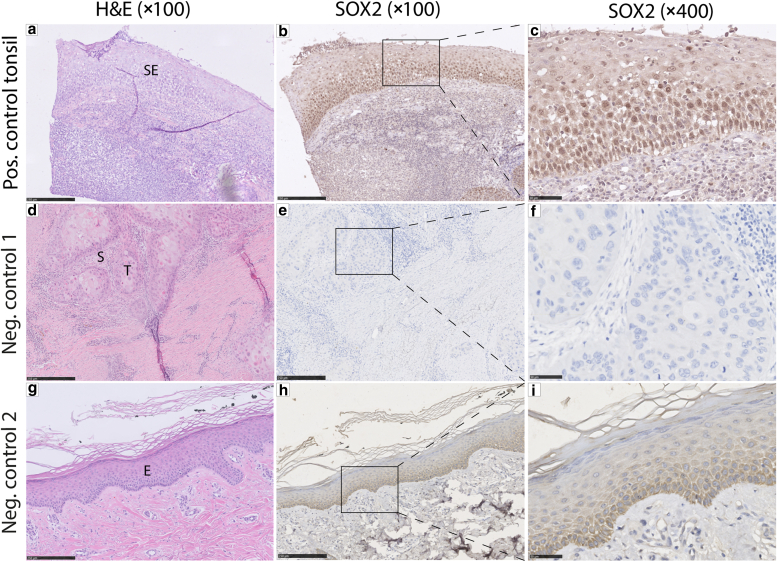
**Positive and negative control for immunohistochemical expression of SOX2.(a)** Representative H&E staining of tonsil tissue selected for the positive control of SOX2 protein expression, covered at the luminal surface with stratified squamous epithelium (denoted as SE). **(b, c)** Diffuse positive nuclear SOX2 expression in the stratified squamous epithelium of the tonsil tissue. **(d)** Representative H&E staining of a well-differentiated EB-cSCC, characterized by sharply demarcated nests of squamous epithelium (denoted as T) surrounded by stroma (denoted as S) selected for the primary antibody omission control (negative control 1). **(e, f)** Primary antibody omission control shows absent (negative) nuclear SOX2 staining in the EB-cSCC (negative control 1). **(g)** Representative H&E staining of a nonatypical epidermis (denoted as E) of a patient without EB, selected for the negative tissue control (negative control 2). **(h, i)** After SOX2 immunohistochemical staining, the epidermis shows absent (negative) nuclear SOX2 expression. An ambiguous cytoplasmic brown staining can be detected, which is not categorized as positive but as nonspecific staining (background staining). Original magnification: (**a, b, d, e, g, h**) = ×100 and (**c, f, i**) = ×400. Bar = (**a, b, d, e, g, h**) = 250 μm and (**c, f, i**) = 50 μm. cSCC, cutaneous squamous cell carcinoma; EB-cSCC, epidermolysis bullosa–associated cutaneous squamous cell carcinoma.

For the validation of the SOX2 antibody, a positive control (human tonsil) and 2 different methods for negative controls were implemented. Antibody validation and selection of positive and negative control tissues were guided using data from the Human Protein Atlas (Uhlén et al, 2015). Regarding the negative controls, a primary antibody omission control and a tissue negative control (nonatypical epidermis of a patient without EB or cSCC) were used (Figure 4).

The sections were scanned with the ×40 objective of the digital slide scanner Hamamatsu Nanozoomer 2.0HT (Hamamatsu Photonic K.K.), and the digital whole-slide images (WSIs) were viewed using Aperio ImageScope (version 12.4.3) (Leica Biosystems) software.

### Analysis of immunohistochemical staining

#### Semiqualitative assessment of SOX2 immunochemical staining pattern

SOX2 staining was first assessed by a dermatopathologist (GD), unaware of the clinical data, using both conventional light microscopy and digital WSI. The evaluation focused on detection of nuclear localization of SOX2 and assessing the staining pattern. SOX2-positive tumor cells were indicated by the presence of brown staining in the nucleus. In addition, the localization of SOX2 expression was described, particularly with regard to its localization (eg, distribution across the TSI). If present, brown signal outside of the nucleus was also described.

The results of the semiqualitative assessment, including the presence of SOX2 protein expression and staining pattern, were described for each section of each experimental group (Table 1). For the collected data, absolute numbers, proportions, and percentages were calculated.

#### Semiautomatic digital scoring of SOX2 protein expression

QuPath (version 0.5.1) was used for the semiautomatic quantification of the immunohistochemical expression of SOX2 in the EB-cSCC and UV-induced cSCC groups (Bankhead et al, 2017). A machine learning–based object classifier was trained using manually selected annotations on the WSI to differentiate histologically distinct tissue classes, including tumor, stroma, immune infiltrates, and nonatypical epidermis. After classifier training, cell detection was performed across the WSIs of each cSCC using QuPath’s Watershed Cell Detection algorithm. The trained object classifier was applied to all detected cells to assign them to the appropriate tissue category. For the assessment of SOX2 expression, a nuclear intensity classifier (nucleus: DAB optical density mean) was used to stratify tumor cell staining, with cells classified as negative (0) or positive (low intensity [1+], moderate intensity [2+], or strong intensity ]3+]) according to predefined intensity thresholds. The H-score was calculated for each WSI, multiplying the staining intensity by the overall percentage of SOX2-positive cells (range 0 [all tumor cells negative]–300 [all tumor cells strongly positive]; H-score = [0 × % negative] + [1 × % low] + [2 × % moderate] + [3 × % strong]).

Statistical analysis and data visualization were performed in R, version 4.5.0 (R Core Team, 2025). Data normality was assessed using the Shapiro–Wilk test, which indicated non-normal distributions. For the percentage of SOX2-positive tumor cells and the H-score, descriptive statistics (minimum, maximum, median, IQR) were calculated separately for the EB-cSCC and the UV-induced cSCC groups. In addition, boxplots were generated to visualize the group-wise distributions.

On the basis of the small sample size and non-normal distribution, the difference in the percentage of SOX2-positive tumor cells and H-score between EB-cSCC and UV-induced cSCC were evaluated using the Wilcoxon rank sum exact test, with statistical significance defined as *P* < .05.

**Figure 5:**
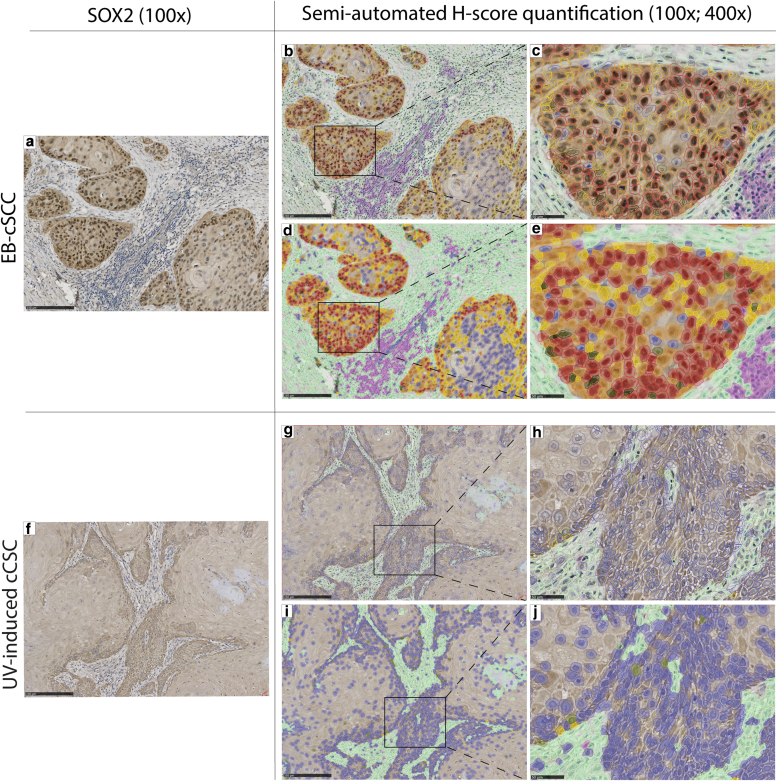
**Semiautomated quantification of SOX2 protein expression in EB-cSCC and UV-induced cSCC with QuPath.(a)** SOX2 staining in EB-cSCC categorized by semiqualitative assessment as ‘positive.’ **(b–e)** Digital masking of the tissue with object and intensity classifier with detecting positive (low intensity [1+], moderate intensity [2+], strong intensity [3+]) or negative (absent nuclear staining [0]) tumor cells. Besides tumor cells, stroma and immune infiltrate is depicted by the trained object classifier. **(f)** SOX2 staining in UV-induced cSCC categorized by semiqualitative assessment as ‘negative.’ **(g–j)** Digital masking of the tissue with object and intensity classifier with same detection settings as in **b–e**. Compared with the EB-cSCC in the UV-induced cSCC, mostly negative tumor cells with absent nuclear staining (0) are detected. There are a few positive tumor cells with low intensity (1+). Original magnification: (a, b, d, f, g, i) = ×100 and (**c, e, h, j**) = ×400. Bar (**a, b, d, f, g, i**) = 250 μm and (**c, e, h, j**) = 50 μm. Colors: Low intensity: yellow; moderate intensity: orange; strong intensity: red; absent nuclear staining (0): blue; stroma: turquoise; immune infiltrate: purple. cSCC, cutaneous squamous cell carcinoma; EB-cSCC, epidermolysis bullosa–associated cutaneous squamous cell carcinoma.

Additional information about the semiautomated analysis with Qupath, including the visualization of the QuPath object and intensity classification and absolute numbers of positive and negative detected tumor cells, is described in Figure 5 and Table 2.

**Table 2:** Additional Data from Semiautomated Analysis for Quantification of SOX2 Protein Expression in EB-CSCC and UV-induced cSCC

Patient ID	Group	Number of Detected Tumor Cells	Number of SOX2-Positive Tumor Cells (Intensity 1+)	Number of SOX2 -Positive Tumor Cells (Intensity 2+)	Number of SOX2-Positive Tumor Cells (Intensity 3+)	Number of Negative Tumor Cells	Percentage SOX2-Positive Tumor Cells (%)	H-score for SOX2 IHC Expression
EB-1	EB-cSCC	187,605	4519	1105	125	181,856	3.06%	3.8
EB-2	EB-cSCC	218,855	22,702	3535	295	192,323	12.12%	14
EB-3	EB-cSCC	419,222	140,852	90,984	8616	178,770	57.36%	83.2
EB-4	EB-cSCC	280,313	3.06%	29,339	4071	181,968	35.08%	48.5
EB-5.1	EB-cSCC	136,366	16,198	3217	309	116,642	14.46%	17.3
EB-5.2	EB-cSCC	39,648	720	80	4	38,844	2.03%	2.3
EB-6	EB-cSCC	66,849	4241	834	58	61,716	7.68%	9.1
EB-7	EB-cSCC	393,067	88,153	45,898	10,132	248,884	36.68%	53.5
EB-8	EB-cSCC	133,120	11,501	2460	93	119,066	10.56%	12.6
UV-1	UV-induced cSCC	13,241	1344	147	6	11,744	11.31%	12.5
UV-2	UV-induced cSCC	128,696	2527	327	14	125,828	2.23%	2.5
UV-3	UV-induced cSCC	175,185	3400	380	15	171,390	2.17%	2.4
UV-4	UV-induced cSCC	13,467	811	44	5	12,607	6.39%	6.8
UV-5	UV-induced cSCC	25,564	1111	84	13	24,356	4.73%	5.2
UV-6	UV-induced cSCC	75,883	4324	575	34	70,950	6.5%	7.4
UV-7	UV-induced cSCC	23,711	1262	103	6	22,340	5.78%	6.3
UV-8	UV-induced cSCC	114,824	2079	300	40	112,405	2.11%	2.4
UV-9	UV-induced cSCC	64,331	775	94	10	63,452	1.37%	1.5
UV-10	UV-induced cSCC	14,717	117	11	2	14,587	0.88%	0.9

Abbreviations: cSCC, cutaneous squamous cell carcinoma; EB, epidermolysis bullosa; EB-cSCC, epidermolysis bullosa–associated cutaneous squamous cell carcinoma; H-score, Histochemical score; ID, identification; IHC, immunohistochemistry.

## Ethics Statement

This study was reviewed and approved by the Board of Directors of the University Medical Center Groningen, Central ethics Review Board non–Medical Research Involving Human Subjects Act studies, and Local ethics Review Board Pathology non–Medical Research Involving Human Subjects Act studies (University Medical Center Groningen Research register number 202000412). Deidentified remaining human material (formalin-fixed, paraffin-embedded tissues) from the Pathology biobank of the University Medical Center Groningen was retrospectively collected for the study, after being no longer required for diagnostic purposes. Therefore, no informed consent of the patients was required, and the study was not considered research subject to the Medical Research Involving Human Subjects Act.

## Data Availability Statement

The data generated and/or analyzed during this study are deposited in the public biorepository DataverseNL and can be used by other investigators. The DOI of the published data is https://doi.org/10.34894/HWOSWP.

## ORCIDs

C. Harrs: http://orcid.org/0000-0002-0034-911X

M. C. Bolling: http://orcid.org/0000-0003-2086-9363

P. C. van den Akker: http://orcid.org/0000-0002-3734-753X

B. Koopman: http://orcid.org/0000-0003-0873-9638

B. Horváth: http://orcid.org/0000-0001-8559-3674

G. F. H. Diercks: http://orcid.org/0000-0001-8053-216X

L. C. van Kempen: http://orcid.org/0000-0003-0646-0705

## Conflict of Interest

The authors state no conflict of interest.
